# A Novel Greenhouse-Based System for the Detection and Plumpness Assessment of Strawberry Using an Improved Deep Learning Technique

**DOI:** 10.3389/fpls.2020.00559

**Published:** 2020-06-03

**Authors:** Chengquan Zhou, Jun Hu, Zhifu Xu, Jibo Yue, Hongbao Ye, Guijun Yang

**Affiliations:** ^1^Institute of Agricultural Equipment, Zhejiang Academy of Agricultural Sciences, Hangzhou, China; ^2^Food science institute, Zhejiang Academy of Agricultural Sciences, Hangzhou, China; ^3^International Institute for Earth System Science, Nanjing University, Nanjing, China; ^4^Key Laboratory of Quantitative Remote Sensing in Agriculture of Ministry of Agriculture P. R. China, Beijing Research Center for Information Technology in Agriculture, Beijing, China; ^5^National Engineering Research Center for Information Technology in Agriculture, Beijing, China

**Keywords:** strawberry detection, deep learning, improved faster-RCNN, plumpness assessment, ground-based imaging system

## Abstract

The automated harvesting of strawberry brings benefits such as reduced labor costs, sustainability, increased productivity, less waste, and improved use of natural resources. The accurate detection of strawberries in a greenhouse can be used to assist in the effective recognition and location of strawberries for the process of strawberry collection. Furthermore, being able to detect and characterize strawberries based on field images is an essential component in the breeding pipeline for the selection of high-yield varieties. The existing manual examination method is error-prone and time-consuming, which makes mechanized harvesting difficult. In this work, we propose a robust architecture, named “improved Faster-RCNN,” to detect strawberries in ground-level RGB images captured by a self-developed “Large Scene Camera System.” The purpose of this research is to develop a fully automatic detection and plumpness grading system for living plants in field conditions which does not require any prior information about targets. The experimental results show that the proposed method obtained an average fruit extraction accuracy of more than 86%, which is higher than that obtained using three other methods. This demonstrates that image processing combined with the introduced novel deep learning architecture is highly feasible for counting the number of, and identifying the quality of, strawberries from ground-level images. Additionally, this work shows that deep learning techniques can serve as invaluable tools in larger field investigation frameworks, specifically for applications involving plant phenotyping.

## Introduction

Strawberry is a perennial root herb and one of the most important berry products in the world (Sønsteby and Heide, 2017). Compared with other types of berries, it has a faster fruit-bearing speed, earlier maturation, smaller plant size, and shorter reproductive cycle. As a cash crop with low investment and high income potential, it has been widely planted all over the world. China has the most abundant wild strawberry resources in the world (103 accessions of wild strawberry genotype), with an annual strawberry production of over eight million tons (Morris et al., 2017). Since the 1980s, through the introduction of hybrid breeding and mutation breeding, many high-yield strawberry varieties have been developed. In strawberry collection, labor costs have always represented by far the highest proportion of the total expenditure (Lu et al., 2017). In the process of strawberry picking and fruit grading, significant numbers of experienced skilled workers are required to perform manual work. Recently, intelligent machines, named strawberry-harvesting robots, have been introduced in the strawberry industry (Bargoti and Underwood, 2017). First, an RGB camera or depth camera is used to capture 2D or 3D images in order to distinguish strawberries from the background. Then, the central control system operates a manipulator to complete strawberry picking based on the results of the fruit location algorithm (Khosro et al., 2018). Numerous studies have achieved the non-destructive recognition and picking of ripe fruit. For example, Ji et al. (2012) developed an automatic visual recognition system for an apple-harvesting robot; they combined a vector median filter with an image segmentation method based on region-growing and color features and finally achieved a recognition success rate of approximately 89%. Furthermore, Liu et al. (2018) proposed a method based on block classification to recognize apples in plastic bags; an edge detection–based watershed algorithm and a support vector machine (SVM) were used to extract color and texture features from blocks, and the recognition of apples in plastic bags was finally realized. Moreover, Savakar and Anami (2009) introduced a classifier named the back-propagation neural network (BPNN) to recognize and classify different kinds of fruit. The obtained average accuracies of 94.1, 84.0, and 90.1% for the selected targets show that the system was efficient and reliable. Additionally, Zhang et al. (2007) proposed a method for the recognition of cucumbers in greenhouses involving the automatic picking of fruits by robots. After successful image preprocessing and network training, the plant images were segmented.

However, the automation of strawberry harvesting presents a number of unique difficulties:

• During the picking process, complete images of strawberries cannot be obtained due to occlusion by leaves and stems.
• Dynamic illumination conditions and surface reflection change the color features of the strawberries and the background.
• Strawberries must be picked at the right time, as they do not ripen significantly once removed from the plant. Any automated system is commanded to pick out all ripe fruit.

Fruit recognition and localization processes play important roles in the development of strawberry-harvesting robots (Gongal et al., 2015). A successful recognition and location model should avoid the misjudgment of strawberries and select suitable fruit for harvesting according to their appearance (Srivastava and Sadistap, 2018). A number of color-pattern recognition methods have emerged in the field of strawberry field-image processing, for example, the K-Nearest Neighbor algorithm, Principle Component Analysis, Linear Discriminant Analysis, and Non-Negative Matrix Factorization (Wu et al., 2017). Luo et al. (2018) designed a vision system to detect cutting points on the peduncles of double-overlapping grape clusters in a vineyard; they used three main steps to detect the cutting point—namely, K-means clustering, edge detection, and geometric information decision-making—and demonstrated the effective practical performance of the system. Wang et al. (2017) combined supervised classification technology with a geometric center–based matching method and built a recognition and matching system for mature litchi fruits. Zhao et al. (2011) proposed an image-based vision servo-control system for harvesting apples. By using an SVM with a radial basis function, the algorithm was able to detect and locate apples in a tree with a successful identification rate of 77%.

Recently, artificial neural networks have been widely used in information processing, pattern recognition, intelligent control, and system modeling, due to their advantages of distributed storage, parallel processing, and self-learning ability (Chaki et al., 2019). Inkyu et al. (2016) presented a fruit detection method using Faster Region–based CNN (Faster-RCNN). They combined information obtained from color and Near-Infrared images, and the final results can be used as a key element for fruit yield estimation and automated harvesting. Furthermore, Madeleine et al. (2016) introduced a novel multi-sensor framework to identify every piece of fruit combined with a state-of-the-art Faster-RCNN detector. They used LiDAR to generate image masks for each canopy so that each fruit could be associated with the corresponding tree. Additionally, Bauer et al. (2019) developed an open-source platform named AirSurf to measure phenotyping information from remote sensing images. A computer vision algorithm was combined with deep learning architecture to realize the quantification of a large number of lettuces using the normalized difference vegetation index (NDVI). However, if the plant varieties and growing conditions are changed, none of the abovementioned methods will be valid. To reliably detect strawberries in the growth stage from RGB images, several changes must be made to the segmentation model, such as changes in the color temperature of the light, changes in reflectance before and after wetting the soil, and lack of typical images of strawberries in universal datasets (e.g., ImageNet) (Deng et al., 2009). Therefore, a robust model must be established, all of whose parameters should be trained using labeled image data.

Fruit quality is a complex parameter that is influenced by the synthesis and action of hormones. The metabolism of sugars and acids is also responsible for the rate of ripening. A small number of researchers have focused on the rapid and non-destructive testing of fruit quality. For example, Zhang et al. (2017) proposed a quadratic polynomial regression model for the assessment of the maturity of peaches based on near-infrared spectroscopy. The experimental results demonstrated a high correlation coefficient between fruit firmness and the index of absorbance difference (IAD). Furthermore, Misron et al. (2017) used a resonant frequency technique to identify the maturity of oil palm fruit bunches; they investigated the resonance frequency of the air coil and tested samples of fresh oil palm fruit bunches. Moreover, researchers have investigated the use of different objective methods to evaluate the internal quality of strawberry; however, the non-destructive and low-cost assessment of the external quality of strawberry (e.g., size and plumpness) is still a challenge due to the unique shape and changing appearance of this fruit.

In this paper, a low-cost image acquisition system was developed in order to obtain images of each strawberry in a greenhouse. In order to avoid interference in the training results due to changing illumination and leaf occlusion, we captured the strawberry images under different illumination intensities. A deep learning model based on transfer learning was used to identify and count the number of target fruits. Additionally, shape information was used to measure strawberry ripeness, which can be useful for studies of precision picking. The experimental results demonstrate that the Improved Faster-RCNN can achieve the suitable recognition and measurement of strawberries in a greenhouse without requiring any prior information. The results of tests on several species of strawberry are presented in this paper, which exemplify the usefulness of the proposed method for the acquisition of phenotypic information for breeding research. From the results, it can be concluded that our method has the potential to assist in the improvement of strawberry varieties as well as in providing decision information for harvesting robots, which is essential in the soft fruit industry. The remainder of this paper is structured as follows: in the materials and methods section, we describe the study area, the image acquisition system, and the data preprocessing process. The images were captured by two near-ground cameras in the greenhouse and were then used to build our original dataset. In the results and discussion section, we analyze the performance of the model by comparison with three other methods. We also provide the results of a method to assess the plumpness of strawberries which involves the application of the minimal external rectangle method. Finally, in the conclusion section, we summarize the significance of the present study and suggest future applications of the method proposed herein.

## Materials and Methods

### Study Area

**FIGURE 1:**
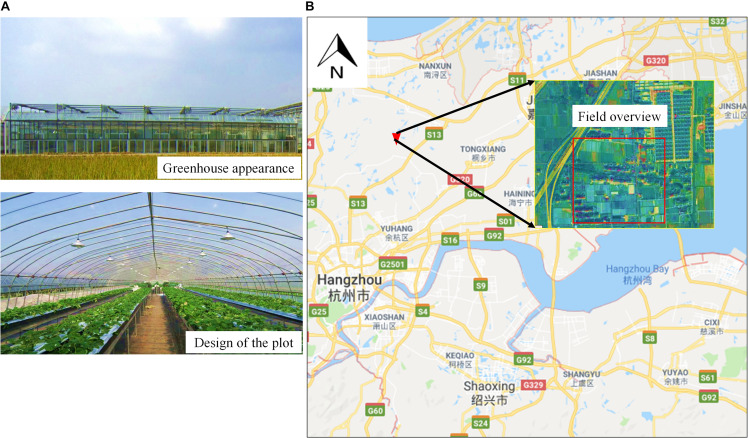
Strawberry field experiment. **(A)** Greenhouse appearance and plot design; **(B)** location of the experimental base.

The strawberry images were captured at the Zhejiang Academy of Agricultural Sciences (ZAAS) Yangdu Scientific Research Innovation Base, Haining County, Zhejiang Province, China (30°27′ N, 120°25′ E). The greenhouse was made of a steel frame and covered by a 0.1-mm-thick polyethylene (PE) film. All strawberries were planted in raised beds placed 0.5 m above the ground which were covered with 0.03-mm-thick PE mulch and were set 0.2–0.3 m apart in rows with a distance of 1.5 m between each row. The soil available phosphorous content was 20 mg kg^–1^, the soil content of rapidly available potassium was 300 mg kg^–1^, and the soil content of alkali-hydrolyzable nitrogen was 300.2 mg kg^–1^. The strawberry samples consisted of three varieties, namely, *Red Cheeks*, *Sweet Charlie*, and *Yuexin*, which are the most widely planted strawberry varieties in Zhejiang Province. There were three plots for each variety, each with an area of 60 m^2^ (30 m × 2 m), and all plants were watered daily (Figure 1).

### Field Experiments and Image Acquisition

**FIGURE 2:**
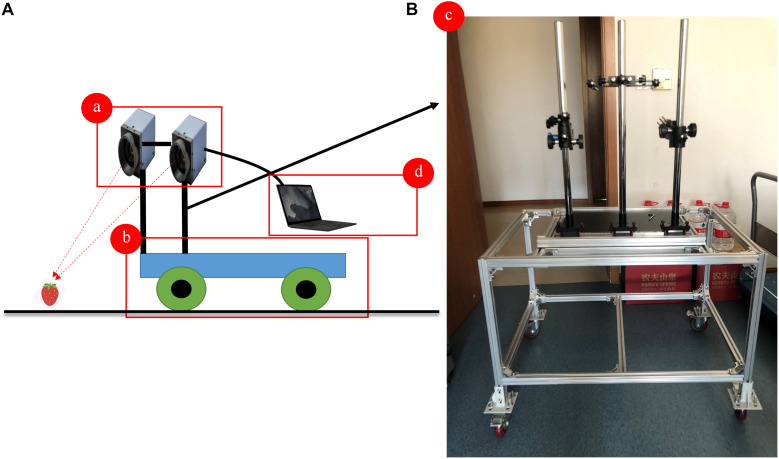
The design of the Large Scene Camera System. **(A)** Design of the system; **(B)** the appearance of the four-wheel mobile platform. (a) MV-SUF1200M-T industrial camera; (b) mobile platform; (c) sensor bracket; (d) storage device.

The workflow of the image acquisition is shown in Figure 1. In the experiment, the images were first captured from the left and right directions by two MV-SUF1200M-T industrial cameras with a 1″-CMOS and a resolution of 12.0 megapixels (MindVision Technology, Co., Ltd., Shenzhen, China), which are collectively called the “Large Scene Camera System.” The system was installed on top of a four-wheel mobile platform and was set to continuous shooting mode to ensure the stability of shooting. For the acquisition of the original dataset, the Large Scene Camera System was positioned 1.5 m above ground level and the system was set looking forward at an angle of 45 degrees to the vertical, resulting in a spatial resolution of 0.05 cm per pixel. The camera aperture was set at an International Standardization Organization (ISO) of 125, and the focal lengths of the cameras were varied from 18 to 55 mm. The two cameras were controlled by an electronic shutter connected to a laptop by a USB 3.0 interface, and the shooting frequency was 1 picture per second. The final original image dataset consisted of 400 images. Then, the panoramic images were generated by calculating the similarity between the corresponding pixels of two overlapping images (obtained synchronously by the two cameras, with each camera pointing in a different direction). The mosaic method consisted of the following three main parts: (1) feature-point extraction and matching based on scale-invariant feature transform (SIFT) (Rublee et al., 2011); (2) image registration; and (3) image fusion. The open-source source codes of the image mosaic algorithm are available at http://www.lfd.uci.edu/∼gohlke/pythonlibs/. The aim of image mosaicking was to obtain more abundant information and then generate combination images with new characteristics. The final digitized panoramic strawberry images were stored in JPEG format on a hard disk (Figure 2).

### Image Preprocessing

#### Noise Reduction

The image acquisition process in the greenhouse was affected by material properties, the transmission medium, and voltage fluctuation in the cameras (Brekhna et al., 2017). This meant that the images contained noise and could not be directly used for interpretation and analysis. Consequently, a median filter with a size of 3 × 3 pixels was used to remove high-frequency noise from the images. This window size was chosen according to the numbers of strawberry-containing pixels. The denoised images were obtained through the integration of the red, green, and blue denoised channels.

#### Data Augmentation

The data augmentation process can be divided into two categories: offline augmentation and online augmentation (Parihar and Verma, 2016). In offline augmentation, the number of enhanced data is changed into the product of the value of augmentation factors and the original dataset. Such augmentation is generally used for small datasets. Meanwhile, online augmentation focuses on “batches” and is often used in training using large datasets. The generalization ability of the model can be improved by increasing the amount of data without changing the image category. There are two main approaches for data augmentation with natural images: geometric transformation and pixel transformation. The most commonly used geometric transformation methods are horizontal flip (or mirror), displacement, tailoring, and rotation. During pixel transformation, researchers often use color jittering and noise augmentation to improve the robustness of datasets. In this paper, the original images were processed using eight methods: brightness augmentation and attenuation, chroma augmentation and attenuation, contrast augmentation and attenuation, and sharpness augmentation and attenuation. In these methods, the brightness, chroma, and contrast of the image are attenuated to 1.2 times that of the original image, the sharpness is enhanced to 2 times that of the original image, and the brightness, chroma, contrast, and sharpness of the image are reduced to 60, 60, 60, and 10% of that of the original image, respectively. Additionally, in order to simulate the noise that the equipment may produce during image acquisition, Gaussian noise with a variance of 0.01 was added to the original images. Through the augmentation process, the dataset was expanded by 10 times, reaching 4000 pictures. In order to ensure the credibility of the training results, images were divided into training (80%) and testing (20%) sets (3200 training samples and 800 testing samples).

#### Annotation and Resizing

**FIGURE 3:**
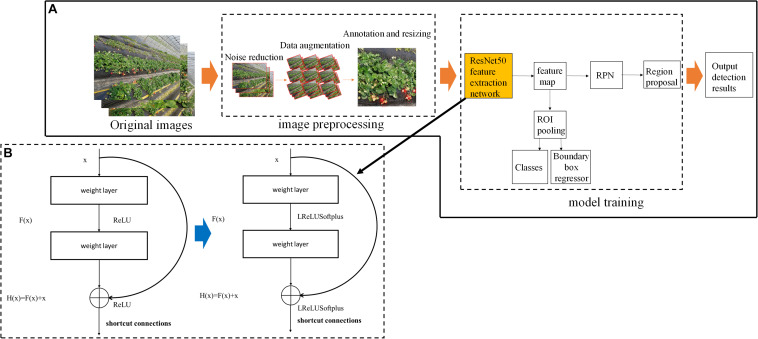
Flowchart of the deep learning algorithm used for strawberry detection. The algorithm consists of two main parts: image preprocessing and model training. **(A)** Architecture of the training models; **(B)** the structure of the residual module in ResNet. RPN, region proposal network; ROI, region of interest.

To verify the performance of the algorithm, all of the images were labeled by three experts. Many open-source tools are available for image annotation, such as Labelme, labelmg, yolo_mark, Vatic, and the Video Object Tagging Tool (Microsoft Corporation). Each of these tools has its own suitable work scenario and task category. Since strawberry recognition in field conditions is a static multi-target detection task, we chose the Video Object Tagging Tool as the labeling tool due to its simplicity and convenience. For each labeled image, there was an additional extensible markup language (XML) file containing the coordinates of the annotated bounding box (see Supplementary Material). Each box was represented as a four-dimensional array—(x_*min*_, y_*min*_, x_*max*_, y_*max*_)—in order to determine its relative position on the graph. Through the above treatment, each image contained about 100–150 boxes, which represents the number of strawberries. Due to limitations in GPU memory and capacity, in this study, images were resized to an appropriate size in order to improve the efficiency of the algorithm and reduce the computational burden. In order to reduce information loss, the images were split into local patches with a size of 256 × 256 pixels (according to the size of the strawberry displayed in the image) (Figure 3A).

### Training and Validation Model

Convolutional neural network (CNN) techniques have gradually replaced traditional machine-learning architectures in target recognition applications since they do not need to consider the effectiveness of feature extraction (McCann et al., 2017). The basic Faster-RCNN (Ren et al., 2017) is mainly composed by three parts: (1) basic feature extraction network, (2) region proposal network (RPN), and (3) fast RCNN, while RPN and fast RCNN share the feature to extract the convolution layer. Based on previous studies of fruit recognition and classification by using Faster-RCNN, we focused on three popular feature extraction architectures, namely, VGG16, ResNet50, and the proposed Improved Faster-RCNN model (combined with ResNet50). This study uses a deep learning method based on image processing technology to detect strawberries in images acquired in a greenhouse and attempts to expand its application to an agricultural intelligent system.

#### The VGG16 Model

The VGG16 architecture follows the same design pattern as the basic VGG architecture, which was proposed by researchers at the University of Oxford, United Kingdom Simonyan and Zisserman (2014). VGG16 is a 16-layer model with input data dimensions of 224 × 224 × 3. Other parameters in the network are as follows: the size of the convolution core is 3 × 3, the pooling size is 2 × 2, the maximum pooling step is 2, and the depths of the convolution layer are 64, 128, 256, 512, and 512, respectively. The convolution blocks in the network consist of 2–3 convolution layers, which can increase the perception ability of the network and reduce the number of parameters. Additionally, the multiple use of the Rectified Linear Unit (ReLU) activation function strengthens the learning ability of the model. In this paper, we experiment with a VGG16 net which contains 13 convolutional layers and in which the output from the convolution layers is a high-dimensional feature map which is sub-sampled by a factor of 16 due to the strides in the pooling layers.

#### The ResNet50 Model

ResNet is a complete network formed by the repeated accumulation of residual learning modules. The original ResNet model was proposed by Dr. He Kaiming of the Microsoft Research Institute (He et al., 2016). The ResNet model has high accuracy and is easy to integrate with other network structures. ResNet allows the original input information to be transmitted directly to the next layer by adding a direct link (known as a highway network) to the network. The introduction of residual modules solves the problem of gradient dispersion and enhances the feature learning ability and recognition performance. The structure of the residual modules is shown in Figure 3B. Set *x* as the input and *F* (x, *W*_1_, *W*_2_) as the output after the convolution between *W*_1_ and *W*_2_. The activation function is set as ReLU, so the final output of the residual module unit *y* can be expressed as follows:

(1) $$y=F\,\left(x,W_{1},W_{2}\right)+W_{s}\,x$$

where *W*_1_ and *W*_2_ represent the weighting parameters to be learned and *W*_*s*_ represents a square matrix that transforms *x* from the input residual module dimension to the output dimension. Two kinds of residual modules are used in the ResNet network structure; one is connected by two 3 × 3 convolution networks in series, while the other is connected by three convolution networks with sizes of 1 × 1, 3 × 3, and 1 × 1, respectively. ResNet can be set to have different numbers of network layers—with the most commonly used numbers being 50, 101, and 152—which are stacked together by the residual modules mentioned above. Considering the training efficiency and the hardware processing capability, we chose a ResNet network with 50 layers for testing, which is known as ResNet50.

#### The Improved Faster-RCNN

The traditional Faster-RCNN achieves target detection using a RPN which can automatically extract candidate regions (Sun et al., 2018). Since strawberry recognition is functionally similar to common target recognition and there is presently no training set related to strawberry recognition, we adopted the already-trained ResNet50 model as the feature extraction network and used the weights obtained from the existing ImageNet universal target training set as the initial value. In supervised learning mode, a large amount of data is needed to train the residual network model. However, at present, there are only a few broccoli image data with labels, which cannot meet the training needs of the depth network model. Therefore, in order to improve the accuracy and generalization ability of the ResNet-50 model, a transfer learning method based on middle-level expression was adopted which combines transfer learning with deep learning. First, ImageNet was used to pre-train the ResNet-50 network in order to allow it to extract image features and the trained network parameters were used as network models. Then, the precise segmentation of broccoli head images was realized by adjusting the parameters of the ResNet-50 network. A three-layer adaptive network was used to replace the full connection layer and the classification layer of the ResNet-50 model, and LReLUSoftplus was adopted as the activation function of the architecture. The computing formulas of ReLU and LReLUSoftplus are given in Eqs 2 and 3, respectively, as follows.

**FIGURE 4:**
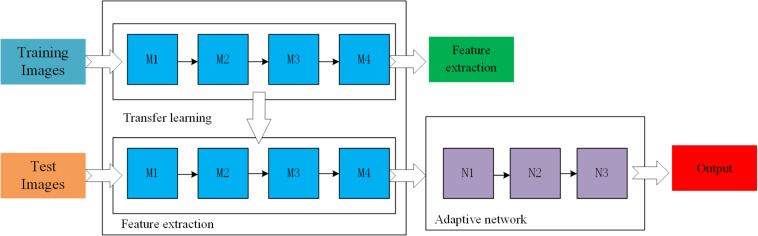
The general scheme of the ResNet based on transfer learning.

(2) f⁢(x)=max⁡(0,x)

(3) $$f\,\left(x\right)=\left\{ \begin{array}{c} \ln\left(e^{x}+1\right)-\ln2,x\geq 0;\\ \text{ax},x<0 \end{array}\right.$$

where *x* indicates the input value and *a* is set as 0.01. A general scheme of the proposed method is shown in Figure 4. M_1_, M_2_, M_3_, and M_4_ are the four residual blocks in the ResNet-50 model while N_1_–N_3_ are the three components of the adaptive network. The number of neurons in layers N_1_, N_2_, and N_3_ is 1000, 256, and 7, respectively, while the activation function of each layer is LReLUSoftplus.

In transfer learning, since the morphology of strawberries is different from that of general targets, the direct application of the model will reduce the accuracy and speed of detection. Therefore, this study improves the area generation network in the Faster-RCNN network framework in the following aspects:

• There are few pictures of strawberries in ImageNet. In this training, a dropout layer was added after the first fully connected layer in order to enhance the generalization ability of the network. The output of a certain proportion of neurons in this layer was randomly inhibited during the training process, and this layer was moved in the testing process. To maintain the corresponding order of magnitude and physical significance of the input in the latter layer, the output value of the above layer was multiplied by the probability of random discarding.

To reduce over-fitting, only one fully connected layer with 2048 output neurons was used to extract the target.

In order to enable a fair comparison between the results of all the experimental configurations, the hyper-parameters for all experiments were standardized as follows: the loss function was set to *dice loss* due to this function’s good performance in dichotomous problems. The base learning rate was 0.001 in the first 3000 iterations and was changed to 0.0005 in the subsequent 2000 iterations. The values of momentum and dropout were 0.9 and 0.5, respectively. The number of epochs was 200, and the batch size was 64.

### Evaluation Index

To evaluate the performance of the proposed method, strawberries that were manually segmented using the Video Object Tagging Tool were compared with the recognition results of the models mentioned in Section “Errors and Limitations.” We used three indexes, namely, Precision, Intersection-over-Union (IOU), and Average Running Time (ART). The computational formulas of these evaluation indexes are shown in Eqs 4–6:

(4) $$A\,c\,c\,u\,r\,a\,c\,y=\frac{T\,P+T\,N}{T\,P+T\,N+F\,P+F\,N}$$

(5) $$I\,O\,U=\frac{C\,a\,n\,d\,i\,d\,a\,t\,e\,B\,o\,x\cap G\,r\,o\,u\,n\,d\,T\,r\,u\,t\,h}{C\,a\,n\,d\,i\,d\,a\,t\,e\,B\,o\,x\cup G\,r\,o\,u\,n\,d\,T\,r\,u\,t\,h}$$

(6) $$A\,R\,T=\frac{N_{t}}{N_{I}}$$

where TP, TN, FP, and FN represent the numbers of true positives, true negatives, false positives, and false negatives, respectively; a true positive represents the correct classification of a region as a strawberry, a true negative represents the correct classification of a region as a background, a false positive means the incorrect classification of a region as a strawberry, and a false negative indicates the incorrect classification of a region as background. IOU is a standard metric that represents the overlap rate between a candidate box and a ground truth bound; the ideal scenario is complete overlap, in which case IOU is equal to 1. In addition to detection accuracy, another important performance index for target detection algorithms is detection speed; real-time detection, which is extremely important for some applications, can only be achieved with high-speed detection. ART represents the time taken by different models to process a certain picture using the same hardware. *N*_*t*_ represents the total running time for all the images, and *N*_*I*_ represents the number of images.

## Results and Discussion

The performance of the newly developed deep learning method was evaluated by conducting several field experiments under changing light conditions and comparing the results of these experiments to the results of the manual measurement of strawberry numbers. All of the recognition models were developed using the open-source TensorFlow software library (Alphabet, Inc., Mountain View, CA, United States), which is a fast software that can be used for deep-learning applications. The experiments were conducted using the Windows 10 operating system on a PC with a four-core 2.3 GHz Intel I5 processor and 4 GB of GPU Memory. The results and comparisons were performed using the Python programming language.

### Strawberry Detection Performance

**FIGURE 5:**
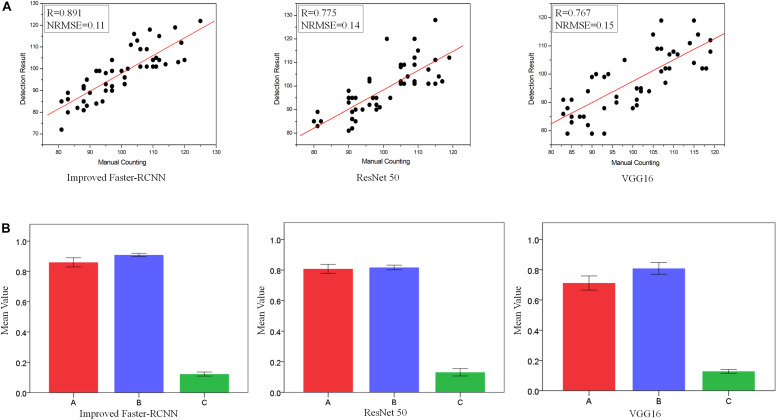
A demonstration of the strawberry detection performance of the introduced model and two other models using the test set. **(A)** Correlations between the number of strawberries identified by manual counting and computer detection; **(B)** comparison of the strawberry detection performances of the introduced model and two other models using the test set. A: Accuracy. B: Intersection-over-Union (IOU). C: Average Running Time (ART). In **(B)**, the red bars represent the mean Accuracy, the blue bars represent the mean IOU, and the green bars represent the mean ART.

In this section, the performance of the strawberry recognition system using the Improved Faster-RCNN is compared with the performances of the other methods described in Section “Training and Validation Model.” Due to the diverse nutritional statuses and genotypes of the plants in the field experiments, a large number of strawberry samples were used to generate the training datasets. Here, we randomly selected 50 pictures from the test set as samples to determine the detection accuracy of different models, and the average number of strawberries in each picture was about 100 (based on manual counting results). Figure 5A shows a comparison between the number of strawberries identified by the Improved Faster-RCNN detection system and that identified by manual inspection (Figure 5A).

As shown in Figure 5A, compared to the other two methods, the Improved Faster-RCNN achieved a higher *R*-value and a lower normalized root mean square error (NRMSE) in the presence of background noise and variable light intensity. Three indexes were used to assess the recognition accuracy, namely, Accuracy, IOU, and ART. The mean and standard deviation (SD) of each evaluation indicator were calculated. The values of these indicators for each of the four recognition methods are shown in Figure 5B. As shown in the figure, the Improved Faster-RCNN achieved a higher Accuracy (0.860) and a lower standard deviation than the Faster-RCNN and the two other classical models. Additionally, the average IOU of the Improved Faster-RCNN was about 0.892, significantly higher than those of the three other models, which shows that this model has a better target extraction ability. Moreover, the Improved Faster-RCNN achieved an ART of 0.158 s, compared with 0.171, 0.163, and 0.182 s for the ResNet50, VGG16, and Faster-RCNN models, respectively. This low ART shows that the Improved Faster-RCNN had a good running efficiency, which can be mostly attributed to the simplification of layers and the reduced number of training parameters. The above results show that the Improved Faster-RCNN can achieve real-time image processing and guarantee the integrity of strawberry detection. Further, the overall F-measure (Hasan et al., 2018) was computed in order to quantify errors. F-measure is a harmonic mean of Precision and Recall which is useful as a measure of the robustness of a model.

As shown in Table 1, compared to the other approaches, the Improved Faster-RCNN had a higher mean quality factor of 0.889. This indicates that the proposed algorithm could accurately detect strawberries in complex scenes.

**TABLE 1:** The overall F-measure of the introduced model and other models using the test set.

Models	F-measure
Improved faster-RCNN	0.889
ResNet50	0.813
VGG16	0.756

**FIGURE 6:**
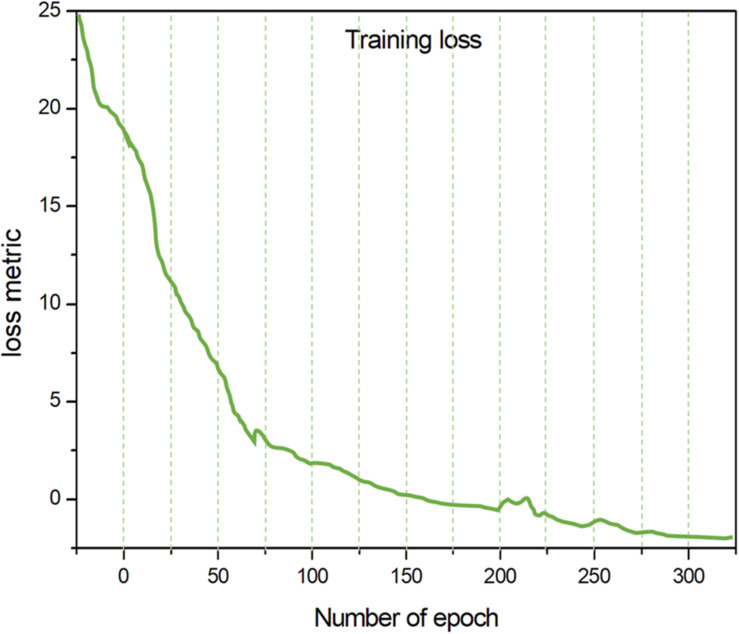
The correlation between the number of epochs and training loss.

In order to further analyze the efficiency of the introduced training models, we determined the loss and error rates during the whole training process. Here, we define an “epoch” as the process of training the model once with all of the image data in the training set (Figure 6).

From Figure 6, it can be concluded that, although the value of the loss metric is initially high, after several rounds of training, the error rate is greatly reduced and the accuracy is significantly improved. No obvious further improvement of accuracy occurred after 200 epochs, and the value of the error rate becomes constant. After around 250 epochs, the benefit of further training appears to be negligible according to the change in the Loss Metric.

### Plumpness Assessment

**FIGURE 7:**
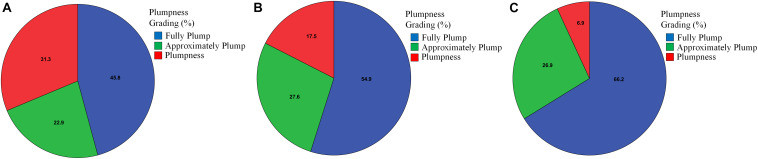
The results of the plumpness assessment for different varieties of strawberry. **(A)** *Red Cheeks* variety; **(B)** *Sweet Charlie* variety; **(C)** *Yuexin* variety.

The assessment of fruit quality is a core requirement for strawberry commercialization. Plumpness—the most important structural trait of fruit quality—can be acquired using the minimal external rectangle method. In this study, the rectangles were represented by labeled boxes. Figure 7 shows strawberries of different shapes with their corresponding external rectangles. The four-dimensional array of each box—(x_*min*_, y_*min*_, x_*max*_, y_*max*_)—was used to determine the location of each strawberry, and the maturity of each strawberry was estimated using the length-to-width ratio of the box, as calculated by y_*max*_ - y_*min*_/x_*max*_ - x_*min*_; the ideal value of this ratio is 1 since the selected varieties are all of globose type (with an aspect ratio very close to 1:1). Through the above process, the length-to-width ratio of each strawberry was estimated, and the strawberries were classified into the following three groups based on this ratio: Plump (0–0.5), Approximately Plump (0.5–0.8), and Fully Plump (0.8–1) (Figure 7).

As shown in Figure 7, the largest average percentage of Plump fruits (31.3%) was detected for the *Red Cheeks* strawberry variety, while the lowest average percentages of Approximately Plump and Fully Plump fruits were also detected for this variety. The second-largest average percentage of Plump fruits (17.5%) was detected for the *Sweet Charlie* variety, while this variety also had the second-highest average percentage of Approximately Plump fruits (27.6%). The lowest average percentage of Plump fruits (6.9%) was detected for the *Yuexin* variety, while the highest average percentage of Fully Plump fruits (66.2%) was also detected for this variety. Thus, it is clear that the *Yuexin* variety has a much faster maturation rate under the current nitrogen application conditions than the other two varieties. Then, the accuracy of our method was tested by comparison with the results of manual grading by three experienced workers. As shown in Table 2, when using the length-to-width ratio, about 85% as many strawberries could be correctly graded compared to the manual grading. Specifically, for the *Red Cheeks*, *Sweet Charlie*, and *Yuexin* varieties, the average prediction Accuracy was 0.879, 0.853, and 0.841, respectively. Thus, the performance of the proposed method can satisfy the requirements for practical use. Further research should focus on the introduction of roundness information or other parameters to build a regression model.

**TABLE 2:** Accuracy of plumpness assessment results for each studied strawberry variety.

	Fully plump	Approximately plump	Plump
Red Cheeks	0.835	0.818	0.896
Sweet Charlie	0.844	0.855	0.862
Yuexin	0.825	0.817	0.894

### Robustness Performance

#### Analysis of Regions With Occlusion or Adhesion

**FIGURE 8:**
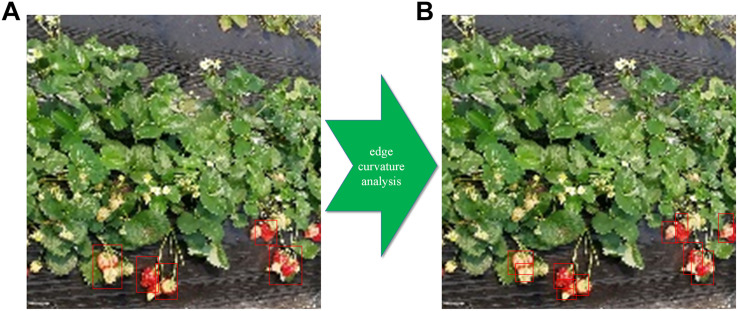
Post-processing of fruit images with occlusion and adhesion using edge curvature analysis. **(A)** Strawberry detection without post-processing; **(B)** detection result after using edge curvature analysis.

This section describes the detection and disconnection methodology for regions with occlusion and adhesion. This methodology consisted of two main steps: first, the RGB strawberry images were converted into binary images, and then shape analysis was performed to determine whether the detection result was isolated or not; second, a watershed-based technique was used to automatically separate the non-independent regions of the binary images. Edge curvature analysis was used to determine whether the fruits were adhered to or occluded (in cases of adhesion or occlusion, the curvature of the edge point at the junction of the fruit edge will change abruptly). The fruit boundaries were accurately determined by removing curvature outliers and by using the watershed algorithm. A visual representation of this algorithm and the adjusted detection results are displayed in Figure 8.

From Figure 8, it can be seen that, in some cases, the strawberries cannot be recognized by the proposed architecture due to occlusion by or adhesion with leaves or other strawberries; in these cases, further processing is required. Thus, in both counting and grading studies, the post-processing methodology for regions with occlusion and adhesion had a significant impact on the final detection accuracy. In order to understand the significance of post-processing using edge curvature analysis, the Accuracy and F-measure were used to express changes in detection accuracy. For detection with the Improved Faster-RCNN network, post-processing increased the Accuracy from 0.860 to 0.878, subsequently increasing the F-measure from 0.889 to 0.901. Therefore, the Accuracy is increased by about 1% by performing this step. Compared with the strategy of simply using a deep learning model, the detection that was performed using the combined workflow was much more strongly correlated with the manual counts for each strawberry variety.

#### Number of Original Images

**FIGURE 9:**
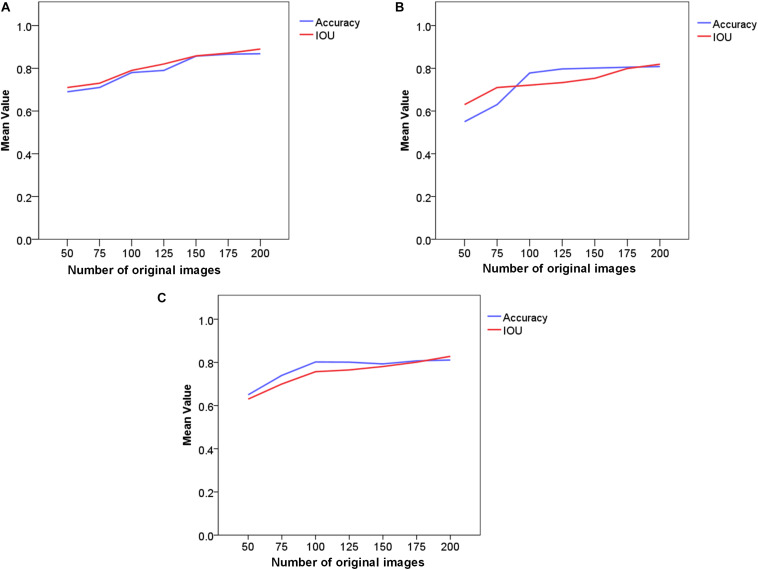
Comparison of the detection performances achieved using different numbers of original images. **(A)** Improved Faster-RCNN; **(B)** ResNet50; **(C)** VGG16. The blue line represents the mean Accuracy and the red line represents the mean IOU.

To examine the utility of using a different number of original images to train the detection algorithm, we varied the number of original images in the learning phase and evaluated the detection performance. All of the images were randomly selected from the whole dataset, and this experiment was repeated five times to take into account the differences between samples (Figure 9).

As shown in Figure 9, using our method, the strawberry detection Accuracy exceeded 0.8 with less than 200 original images. This is an acceptable result considering the complex environment, which involved, e.g., dynamic light intensity, different vegetation canopy reflectances, shadows, and mutual occlusion between leaves and fruits. Furthermore, the introduced deep learning architecture achieved the highest IOU of all of the three approaches, which shows the high training efficiency of the presented method for the small-scale dataset. When less than 200 training images were used, the average value of IOU was about 0.89, which shows the significant potential of the proposed approach in real-world applications. The results also show initial benefits with a larger amount of data; however, these benefits quickly diminish with an increasing number of training images, with a difference in Accuracy of less the 0.05 in the last three compared phases.

#### Light Intensity

**FIGURE 10:**
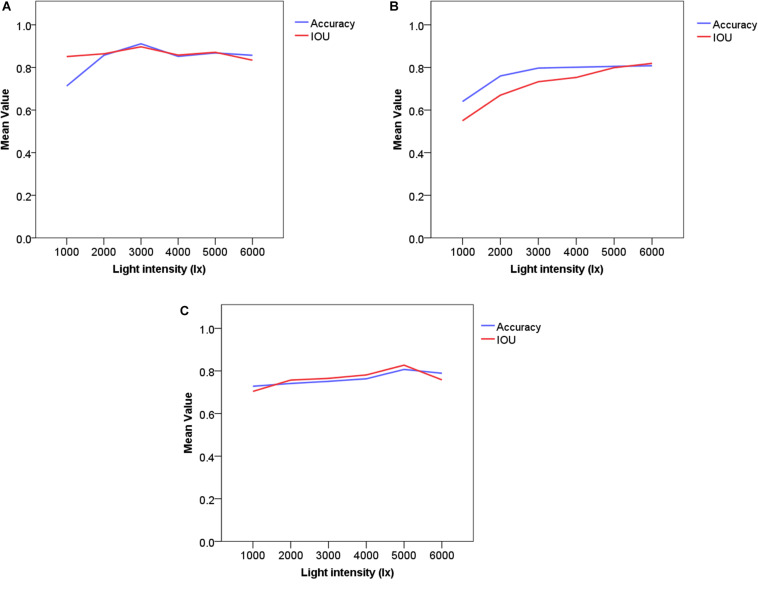
Comparison of training performances obtained using images acquired under different light intensities. **(A)** Improved Faster-RCNN; **(B)** ResNet50; **(C)** VGG16. The blue line represents the mean Accuracy and the red line represents the mean IOU.

Illumination conditions change frequently in outdoor environments. To determine the ability of the Improved Faster-RCNN architecture to detect strawberries under different light intensities, the architecture was tested using images obtained under different illumination conditions. For each illumination level, the Matlab software (MathWorks, Natick, MA, United States) was used to tune the brightness component of each test image, and the test set was separated into five clusters from 1000 Lx to 6000 Lx (Figure 10).

From Figure 10, it can be seen that the Improved Faster-RCNN is less sensitive to changes in outdoor illumination than the other models. Both of the two low-light conditions were associated with low Accuracy and IOU values, while the normal lighting condition was associated with higher Accuracy and IOU values than the other lighting cases. For light intensities of 4000, 5000, and 6000 Lx, the Accuracy values were 0.852, 0.868, and 0.857, respectively. To summarize, the Improved Faster-RCNN can adapt to different light conditions. This will allow us to use this model to perform different tasks, such as the detection of spikes or panicles in field environments.

### Errors and Limitations

#### Errors

As stated in Section “Robustness Performance,” the method employed in the present study contains some errors. These can be attributed to (1) mutual occlusion between strawberries and leaves and (2) irregular fruit shape and changing surface texture. Overlapping is usually detected using the non-maximum suppression (NMS) algorithm, which predicts the number of fruit targets based on the statistical distribution of candidate boxes and interactive higher-order features in dense regions. Introducing such techniques may allow the identification of overlapping objects. The shape and texture of fruits differ between different varieties of strawberries and between individual fruits of the same variety. As shown in Figure 5, the very different degrees of plumpness which were detected between different varieties of plants confirmed that the proposed model is particularly robust when dealing with different varieties of strawberry. However, the differences in shape and texture between different varieties will make the labeling procedure more tedious and prone to errors; this will likely lead to annotation errors, which in turn will cause many false-positive identifications.

#### Limitations

Separating plants from background is a common challenge in plant detection and evaluation, especially in natural environments. Recent studies which used color or texture information for plant detection were able to successfully distinguish between fruit and growing plants and thereby achieved highly accurate yield estimations. However, there are a number of limitations to the method presented in the current study which must be addressed in future work. The first limitation of the present study is that, when a set of images taken by a near-ground vehicle is used for plant detection, it is important to account for the angle at which the images were acquired; for example, many characteristics of strawberries cannot be observed from oblique images. Thus, future plant-detection efforts should attempt to obtain images from different angles.

The second limitation is that we are facing up a time-consuming manual effort. Some of the annotation errors can be reduced by applying a voting strategy among the different human labelers; however, this would greatly increase the labor cost. Automatic annotation could provide a less expensive means to reduce labeling errors; however, manual checking would still be required to ensure labeling accuracy.

### Future Work

In this work, a strawberry detection and grading system based on near-ground image data using a state-of-the-art framework is presented. The use of CNNs has dramatically improved the performance of fruit detection compared with other traditional approaches. However, some issues need to be addressed in future studies in order to reduce the training complexity and improve the environmental robustness of fruit detection based on convolutional neural networks. First, a larger sample set should be introduced to the presented model to solve the problem of over-fitting with fewer labeled examples. Second, the ratio between the size of the test set and the size of the training set should be varied to ensure that the introduced model achieves relatively high accuracy even in extreme cases.

## Conclusion

The continuous increase in labor cost is increasing the expenditure requirements within the strawberry industry. Therefore, many producers are looking toward technological solutions such as the automated harvesting and grading of fruit. In this research, we developed and tested a new approach for the detection of strawberries in greenhouses using a deep convolutional neural network. The results showed that the proposed approach successfully detected strawberries with an average Accuracy of 86.0% and an average IOU of 0.89. Additionally, this study developed a process for the assessment of fruit maturity based on the minimal external rectangle. By varying the number of training images and varying the light intensity, it was shown that the proposed model is capable of accurately detecting strawberries in a complex and changing imaging environment. Furthermore, it is shown that the proposed algorithm has a higher accuracy and efficiency than the ResNet50 and VGG16 models. Thus, this methodology could be applied in multitasking automatic or semi-automatic imaging systems which are used inside greenhouses. Depending on the systems’ requirements, the algorithm could be adjusted without intensive skilled human intervention. The future development of the proposed method promises a clear advantage over other color-based approaches and traditional machine learning approaches as the method can be applied to other types of applications and is not limited to greenhouse environments.

## Data Availability Statement

The raw data supporting the conclusions of this article will be made available by the authors, without undue reservation.

## Author Contributions

CZ developed the detection and measurement algorithm, implemented the existing methods for comparison purposes, conducted the comparisons, obtained the results, and drafted the manuscript. GY developed the detection algorithm. HY supervised the project, identified the main goals of the project, and contributed to writing the manuscript. JH and JY prepared the images for the research. ZX provided expert supervision for marking the ground truth. All authors read and approved the final manuscript.

## Conflict of Interest

The authors declare that the research was conducted in the absence of any commercial or financial relationships that could be construed as a potential conflict of interest.
